# Applying network analysis to explore family and peer influence on diet and health in households of Singaporean young adults: findings from an online network survey

**DOI:** 10.1017/S136898002510133X

**Published:** 2025-11-14

**Authors:** Shahmir H. Ali, Kimberly Mei Yi Low, Cindy Mei Jun Chan, Ian Yi Han Ang

**Affiliations:** Saw Swee Hock School of Public Health, National University of Singapore, Singapore

**Keywords:** Social network analysis, Social network, Dietary behaviour, Family influence, Peer influence, Health behaviour, Noncommunicable disease

## Abstract

**Objective:**

The complexity and nuance of how social networks shape dietary behaviours and health dynamics remain underexplored, particularly in collectivist societies where family and peer relationships strongly impact health. This study applies social network analysis to examine these dynamics in Singapore.

**Design:**

An online household survey of young adults (age 21–35) and family (21+) assessed the consumption of healthy food groups (fruit, vegetable intake), unhealthy food groups (fast food, snack consumption) and social network characteristics (interaction frequency, emotional closeness, shared meals and perceived health influence). Data were analysed using network analysis, mixed regression models and generalised estimating equations.

**Setting:**

Online Singaporean household survey.

**Results:**

Among 116 participants from thirty-six households, 345 unique individuals and 1145 dyadic relationships were identified, with networks averaging 9·7 nodes (sd: 4·7) and 33·2 edges (sd: 27·3). Mutual health influence was strongest in spousal (*β* = 0·89, 95 % CI: 0·42, 1·35) and intergenerational ties (older-to-younger: *β* = 0·62, 95 % CI: 0·29, 0·94; younger-to-older: *β* = 0·36, 95 % CI: 0·03, 0·68) and associated with emotional closeness (*β* = 0·38, 95 % CI: 0·30, 0·46) and shared meals (*β* = 0·43, 95 % CI: 0·36, 0·49). Greater family health effort correlated with lower snack (Adjusted Odds Ratio [AOR]: 0·50, 95 % CI: 0·29, 0·85) and fast-food consumption (AOR: 0·41, 95 % CI: 0·22, 0·77), while higher perceived family health associated with increased snack intake (AOR: 3·21, 95 % CI: 1·58, 6·52). Frequent meals with friends associated with lower fast-food intake (AOR: 0·50, 95 % CI: 0·30, 0·84), but no associations with fruit or vegetable intake were found.

**Conclusion:**

Findings highlight intergenerational and spousal ties as key health influencers, particularly through shared meals, and the complex role of social networks in shaping diet. Analyses suggest network-based interventions may be more useful in reducing unhealthy rather than promoting healthy eating behaviours.

Noncommunicable diseases, such as heart disease, diabetes and cancer, are a major global public health concern^(1)^, driven largely by preventable behavioural risk factors, including unhealthy diets, which alone accounted for 22 % of all deaths globally in 2017^(2)^. Singapore exemplifies this trend, with noncommunicable disease contributing to 74 % of deaths in 2022, slightly exceeding the global average^(3)^. A closer look at Singaporean dietary patterns reinforces these trends, with fruits and vegetables consumed significantly less frequently than beverages, grains, or grain-based products^(4)^. High prevalence of snacking behaviours and frequent consumption of processed foods – including weekly fast food intake by 1 in 5 Singaporeans^(5)^ and regular consumption of confectioneries and chips by 60 % of the population^(6)^ – have likely contributed to rising fat and sodium intake in recent years^(7)^. Notably, these behaviours are shaped not only by individual preferences and environmental factors but also by the social contexts in which they occur, including relationships with family and friends. While significant research has examined individual and environmental determinants of health behaviours, social networks (comprising both familial and peer relationships) remain an underutilised resource for addressing noncommunicable disease risk factors^(8)^. The mutual influence of social network members on each other’s health, particularly in the context of dietary behaviours, offers a promising but understudied area for health promotion.

Social Network Theory and Family Systems Theory provides valuable frameworks for understanding how social networks influence health behaviours. Social Network Theory emphasises the structure and dynamics of relationships within a network, highlighting how individuals’ behaviours are shaped by the norms, resources and information circulating within their social ties^(9)^. Family Systems Theory complements this by focusing on the unique interdependence within families, where changes in one member’s behaviour can ripple through the entire unit^(10)^. Both theories point to key mechanisms, such as role dynamics, feedback loops and shared norms, as central to understanding health influence within networks. For example, certain individuals within a network, whether family members or friends, may take on roles like coordinating health-related activities or sharing knowledge about healthy behaviours^(8,11)^. These roles, along with positive feedback loops (such as collective efforts to adopt healthier eating habits or engage in physical activity), can promote sustained behaviour change. Conversely, shared unhealthy routines can perpetuate risk behaviours, underscoring the need for deliberate interventions to shift network norms^(12)^.

Real-world evidence echoes the aforementioned, increasingly demonstrating the critical role of social networks in shaping health behaviours across various domains^(8,11)^. Past studies have affirmed that both family and friends can provide social support, serve as role models and reinforce shared norms, thereby encouraging health-promoting behaviours^(8,11)^. Among young adults, family and peer influences shape health behaviours by reinforcing shared norms, expectations and routines around daily habits, diet, social interactions and lifestyle choices^(13)^. Research in Singapore also highlights how strong work demands, family obligations and social norms around eating together shape young adult lifestyle behaviours^(14)^. However, the structure and characteristics of social networks (such as density, size, diversity and centrality) play a crucial role in shaping health behaviours and adherence to change efforts. These factors influence interaction frequency, social tie strength and emotional closeness, which, in turn, drive behaviour change through mechanisms such as observation, social comparison, conformity and verbal influence^(11,15)^. In conjunction with social contexts such as communal meals or shared recreational activities, the above factors interplay to provide increased opportunities for families and friends to positively influence each other’s health while promoting overall enjoyment^(16)^. Similarly, unhealthy dietary habits can spread through the same social mechanisms and settings, reinforcing norms that normalise and increase acceptance of purchasing and consuming unhealthy foods^(17)^. Regardless of outcome, much of the existing research focuses on either family or peer influence in isolation, leaving a gap in understanding the combined and comparative effects of these relationships on health behaviours^(18)^.

Cultural values, particularly individualism and collectivism, significantly shape how these social networks operate^(19)^. In collectivist societies, where interdependence and group cohesion are emphasised, social networks often prioritise shared responsibilities and collective decision-making^(19)^. For instance, family members and friends in collectivist cultures may work together to support communal health goals, such as preparing meals that align with shared dietary needs^(20)^. Conversely, in individualistic societies, relationships may centre more on supporting personal autonomy, with health behaviours viewed as individual responsibilities^(21)^. While qualitative studies have emphasised the significant influence of social networks on health behaviours in Asian communities^(22,23)^, particularly in comparison with Western contexts, a gap in quantitatively understanding how collectivist values influence health behaviours within both familial and peer networks prevails.

Social network analysis (SNA) offers a powerful methodological approach to address these gaps. By mapping the structure of relationships within both familial and peer networks, SNA can identify key influencers, patterns of behaviour transmission and the density and strength of social connections^(24)^. This approach enables researchers to examine how social structures, norms and interactions influence health outcomes^(25)^. While SNA has been widely applied in areas such as tobacco use, sexual health and alcohol consumption, its application to preventive health behaviours, particularly dietary practices within social networks, remains limited – especially in non-Western populations^(26,27)^. While reviews show that SNA methods have been occasionality applied to assess similarity in BMI, dieting, body image concerns and fast food intake within youth friendship networks^(28)^ or associations between friends’ specific eating habits, energy intake and popularity^(29)^, most studies focus on general peer ties and aggregated measures rather than detailed egocentric structures, with little evidence for dietary network dynamics of adults or among family members. Expanding the use of SNA to explore how social networks influence health behaviours can inform culturally tailored interventions that leverage both family and peer relationships.

This study aims to examine the importance of social networks members, including family and friends, in shaping how young adults and their households in Singapore perceive and contribute to each other’s health, with a particular focus on healthy and unhealthy dietary practices. By employing SNA, the study will explore how social dynamics and cultural values, particularly individualism and collectivism, shape perceived importance in a friend or family’s health and patterns of influence related to behaviours such as vegetable and fruit consumption, frequency of eating fast food, snack intake and shared meals. The findings are expected to provide critical insights into how social networks function as systems of mutual health influence, offering evidence to inform interventions that harness the power of relationships to promote healthier behaviours and address noncommunicable disease risk factors in culturally diverse settings. It is hypothesised that higher collectivism, lower individualism, more frequent interaction and stronger emotional closeness will be associated with perceiving oneself as playing a more important role in network members’ health. Additionally, better average network health and higher effort put into health within family and peer networks are hypothesised to be associated with healthier individual eating behaviours.

## Methods

### Recruitment

Data were collected from the Supporting Household heAlth through familY-led Promotion (SHAYP) study, a pilot intervention where Singaporean young adults took a co-created online course to help their families reduce sodium intake. Eligible participants were 21- to 35-year-old English-speaking Singaporean citizens or Permanent Residents with 1–3 family members with whom they regularly ate, cooked or grocery shopped. Recruitment occurred via social media and university groups, after which participants invited 1–3 English-speaking family members (aged 21+) to join. Family members could include parents, siblings, grandparents, aunts/uncles, cousins, spouses and in-laws. All participants completed a 30-minute pre-intervention survey via Qualtrics, which serves as the focus of this study. Although the larger SHAYP study targeted young adults, this analysis includes all surveyed participants, regardless of age. The study was approved by the National University of Singapore Institutional Review Board.

### Measures

The pre-intervention survey, which was pre-tested with ∼8 pilot participants from an earlier phase of the study to ensure clarity and appropriateness, included questions related to participants’ demographic background, diet and social networks. Demographic questions included age, sex, race/ethnicity, household income, type of housing (i.e. public housing through the Singapore Housing Development Board, including number of rooms or private housing), marital/relationship status, self-rated health status (from 1, poor to 10, excellent) and self-rated effort put into following a healthy lifestyle (from 1, no effort, to 10, lot of effort). Additionally, participants were provided a four-item, nine-point horizontal individualism and four-item vertical collectivism questionnaire, which was summated to create a score from 4 to 36 for each construct^(30)^, with a higher score reflecting higher individualism/collectivism. Although these items have not been specifically validated in Singapore, they have been applied or validated in Asian and South/Southeast Asian samples with comparable cultural contexts (e.g. Vietnam, China and India) to examine cultural orientations linked to health and social behaviours^(31,32)^. In the current sample, the items demonstrated acceptable to good internal consistency (Cronbach’s alpha = 0·78 for individualism; 0·84 for collectivism). For multivariable analyses, scores were dichotomised into above/below median values (twenty eight for both scales).

Dietary healthfulness was assessed using a four-item, seven-point questionnaire adapted from a previously developed dietary survey for Singaporean adults^(33)^. The questionnaire focused on key food groups representing common healthy and unhealthy dietary choices in Singapore. Participants reported how frequently (none to 4+ times per day) they consumed the following foods in the past 7 days: vegetables (e.g. broccoli, carrots, mushrooms and lentils/*dhal*), fresh fruits (e.g. apples, oranges, bananas and grapes), fried or sweet snacks (e.g. donuts, *you tiao*, chips, cakes, chocolate, sweets and ice cream) and fast food (e.g. burgers, pizza and french fries). Although the initial, short questionnaire was developed for children, other dietary research on common foods consumed in Singapore was used to ensure that the food groups aligned with those commonly consumed among adult Singaporeans as well^(34)^. For analysis, responses were dichotomised based on dietary recommendations. Vegetable consumption was categorised as ≥ 2 times per day *v* less, in line with Singapore’s Health Promotion Board guidelines^(35)^. While the same recommendation applies to fruit intake, fewer participants met this threshold (8 out of 116), so fruit consumption was categorised as ≥ 1 time per day *v* less to maintain statistical power. Given recommendations to limit processed, oily and sugary foods, fast food consumption was dichotomised as none *v*. any^(36)^. Because very few participants (8 out of 116) reported zero snack consumption, snacks were classified as ≤ 3 times per week *v*. > 3 times per week for meaningful comparison.

Participants were then asked to complete a social network questionnaire by first identifying 2–10 of the most important people in their lives, which could include people that they were physically or emotionally close to and/or frequently interacted with. Each important person could be identified based on their relationship to the participant, initials, or a pseudonym (e.g. ‘mother,’ ‘oldest brother’ or ‘auntie AZ’). For each identified important person, participants were asked to describe their relation to them (i.e. friend, family or other), their sex, approximate age, perceived health status and effort put into following a healthy lifestyle (from 1 to 10 using the aforementioned prompts). Next, participants were asked to rank how strongly they agreed (from strongly disagree or not at all, scored as 0, to strongly agree or all the time, scored as 9) with the following statements, when applied to their relationship with the important person: they frequently interacted with each other, had a close emotional relationship, frequently ate with each other, the participant played an important role in the important person’s health and the important person played an important role in the participant’s health. Next, participants were similarly asked to rank how strongly these statements applied to the relationships between the different important people identified by the participant.

### Analysis

Prior to analysis, the number of unique important people (i.e. ‘nodes’) reflected across the social network surveys of the participating young adults and their 1–3 family members were first identified to develop a list of nodes for a family-specific social network analysis. A node is the basic unit of analysis in SNA, representing an individual actor within a network. Common nodes identified by multiple participants within a family unit were renamed for consistency (notably for family relations) to reflect their relationship to the main young adult participant; for example, a young adult may have identified their ‘father’ as an important person, their participating mother may have identified her ‘husband’, and their participating grandmother may have identified her ‘son’ – this common node would be renamed to ‘father’. If there was any uncertainty when trying to identify unique people across these lists, a member of the research team would contact participants to clarify any such ambiguity.

Next, all the unique dyadic interaction data (i.e. ‘edges’) among all identified nodes were consolidated for each family unit to create a list of all dyadic interactions (i.e. ‘edgelist’). In SNA, an edge represents a connection or relationship between two nodes within a network, while an edgelist is a data structure representing all the different edges reflected within a network. Some edges reflected undirected relationships between nodes (e.g. frequency of eating together), while others reflected directed relationships (e.g. importance in each other’s health), comprising of a ‘source node’ (the starting point of the relationship, or transmitter) and ‘target node’ (the end point of the relationship, or recipient). For example, if a participants’ sister and brother were reflected in a network map, they were asked to indicate how important their sister was (source node) in their brother’s health (target node) and vice versa, resulting in two data points. In the case of common nodes, where multiple participants provided data on the same dyadic interaction (e.g. young adult describing the relationship between their father and younger sister, and the participating mother describing the relationship between her husband and younger daughter), the ranks/data corresponding to these dyadic relationships were averaged in the edgelist.

Edges were also classified into the following categories: intergenerational family relations, intragenerational family relations, spouse or partner relations and friend relations. Intergenerational family relations were those which crossed at least one generation; for example, between a grandparent and a parent, a parent and a young adult or a young adult and a niece. Intragenerational family relationships were those between two individuals of the same generation: for example, between a brother and a sister, a mother and an aunt or grandparent and a granduncle. In Singaporean society, godparents can exhibit dynamics similar to a close family member^(37)^ and were thus classified in the same generation as parents or aunts/uncles. Finally, relations between spouses and partners were also disaggregated in analysis; for example, between a mother and father, young adult and girlfriends/boyfriends or brother and sister-in-law. The inclusion of unmarried partnered relationships in this category reflects the reality that they often mirror the social behaviours of married couples^(38)^, especially as societal attitudes towards marriage evolve, particularly among young adults. In Singapore, growing acceptance of unmarried relationships, such as cohabitation^(39)^, highlights the challenge of grouping these partnerships with intragenerational family or friend relationships. Such relations were determined by the name given to the node (e.g. ‘Husband’), and participants were consulted in situations of relational ambiguity. Friend relations were those that involved any non-family relation.

Descriptive analyses were first conducted on all unique dyadic interactions of participants and other identified nodes in their social networks, which included examining the average frequency of interacting, emotional closeness, frequency of eating together and the average importance in the health of target nodes; differences across different relationship types were examined. The assortativity of nodes within each family network (i.e. the degree of association of nodes with similar characteristics) was also analysed, focusing on similarities in age, sex, self-rated health and effort put into health and weighted by frequency of eating together and importance in each other’s health.

Multivariable analyses were conducted to identify node and edge characteristics associated with an increased importance in a target node’s health. Although traditional ego-centric network analyses rely on the assumption that there are no overlaps among the personal networks of different participants, this was not the case in our study (where multiple members of the same family unit had multiple common nodes and common edges). Thus, multivariable analyses were conducted using a method of cross-classified multilevel models that accounts for overlapping nodes and nesting within common family units^(40)^. Specifically, multilevel mixed effects models were constructed which adjusted for the random effects of each node and family unit. Models examined how interaction characteristics (interaction frequency, emotional closeness and frequency of eating together) and the age, sex, self-rated health and effort put into health of both the source and target node associated with an increased importance in the target node’s health.

To explore the association between overall network characteristics and dietary behaviours, we constructed a series of logistic regression models through generalised estimating equations to examine how average perceived health, average effort put into health and average frequency of eating together with network members were associated with the odds of more frequently consuming vegetables, fruits, fast food and snacks. Analyses also calculated network-level averages separately for family members (excluding spouses/partners) and friends within participants’ networks. However, as only fifty-eight out of 116 participants included a spouse or partner in their network, sub-group analyses for this category were not feasible. For this analysis, generalised estimating equations was chosen for its robustness in estimating population-average effects, accounting for correlation within families without requiring estimation of random intercepts, thereby improving model convergence (especially considering sample size constraints for this analysis), thus making it a more appropriate and efficient choice. Analyses were conducted using R (version 4.3.0) using the lmer, geepack and igraph packages to support multi-level and social network analyses.

## Results

Overall, 345 unique individuals and 1145 dyadic relationships were identified in the social networks of the 116 participants surveyed (thirty-six households) (Table 1). Surveyed participants included thirty-six young adults and eighty of their family members, a majority of whom were aged 19–39 (61·2 %), female (56·9 %) and Chinese (76·7 %). Moderately high individualism (27·49 out of 36, sd: 4·99) and collectivism (27·21 out of 36, sd: 6·28) was reported. The average self-rated health of the sample was moderately high (7·10 out of 10, sd: 1·64), with effort put into personal health somewhat less (6·61 out of 10, sd: 1·95). Survey participants cumulatively identified 226 other individuals in their social network; no major differences in sex, health status and effort put into health were reported between participants and others in their network, although a greater proportion of individuals aged 60 or older were reflected among others in the network (27·4 % *v*. 8·6 %, *P* < 0·001). Overall, 27·6 % of participants reported consuming the recommended intake of two servings of vegetables each day, while less reported consuming at least two servings of fruits (6·9 %). Moreover, while 32·8 % of participants reported consuming no fast food in the last week, only 6·9 % reported consuming no snacks.

**Figure 1.**
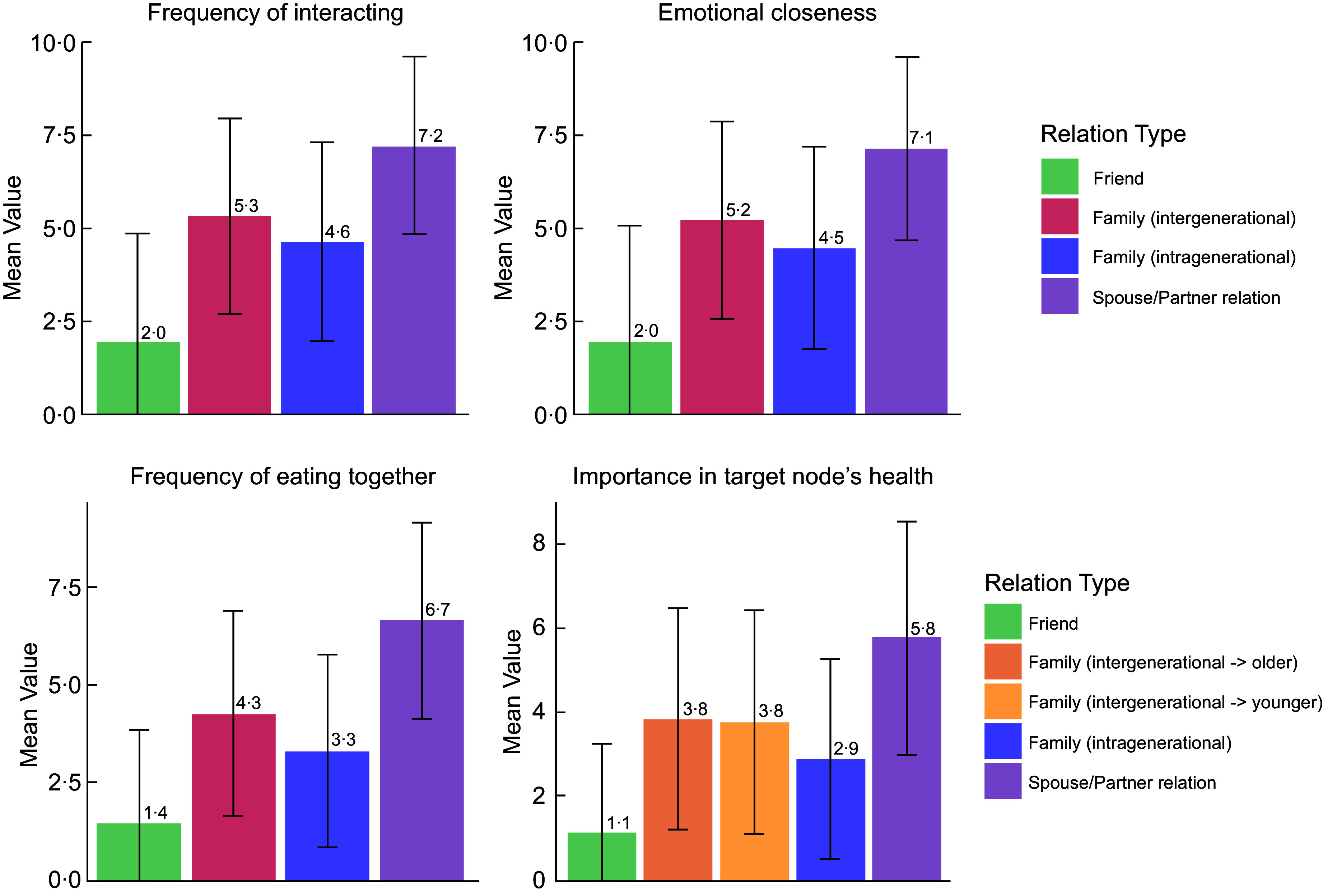
Characteristics of dyadic-directed relations identified among important people in participants’ social networks (*n* 1145).

The 1145 unique dyadic relationships across the thirty-six household social networks analysed included 456 intergenerational family relations, 228 intragenerational family relations, 69 spouse/partner relations and 392 friend relations. Descriptive analyses of these relationships (Figure 1) revealed a high degree of interaction frequency and emotional closeness in spouse/partner (7·23, 7·11) and intergenerational family relations (5·34, 5·20), especially in comparison with friend relations (1·95, 1·95). Moreover, the greatest importance in a target node’s health was observed in spouse/partner relations, followed by intergenerational family relations (3·82 when the target node was in an older generation relative to the source node and 3·76 when the target node was in a younger generation).

**Figure 2.**
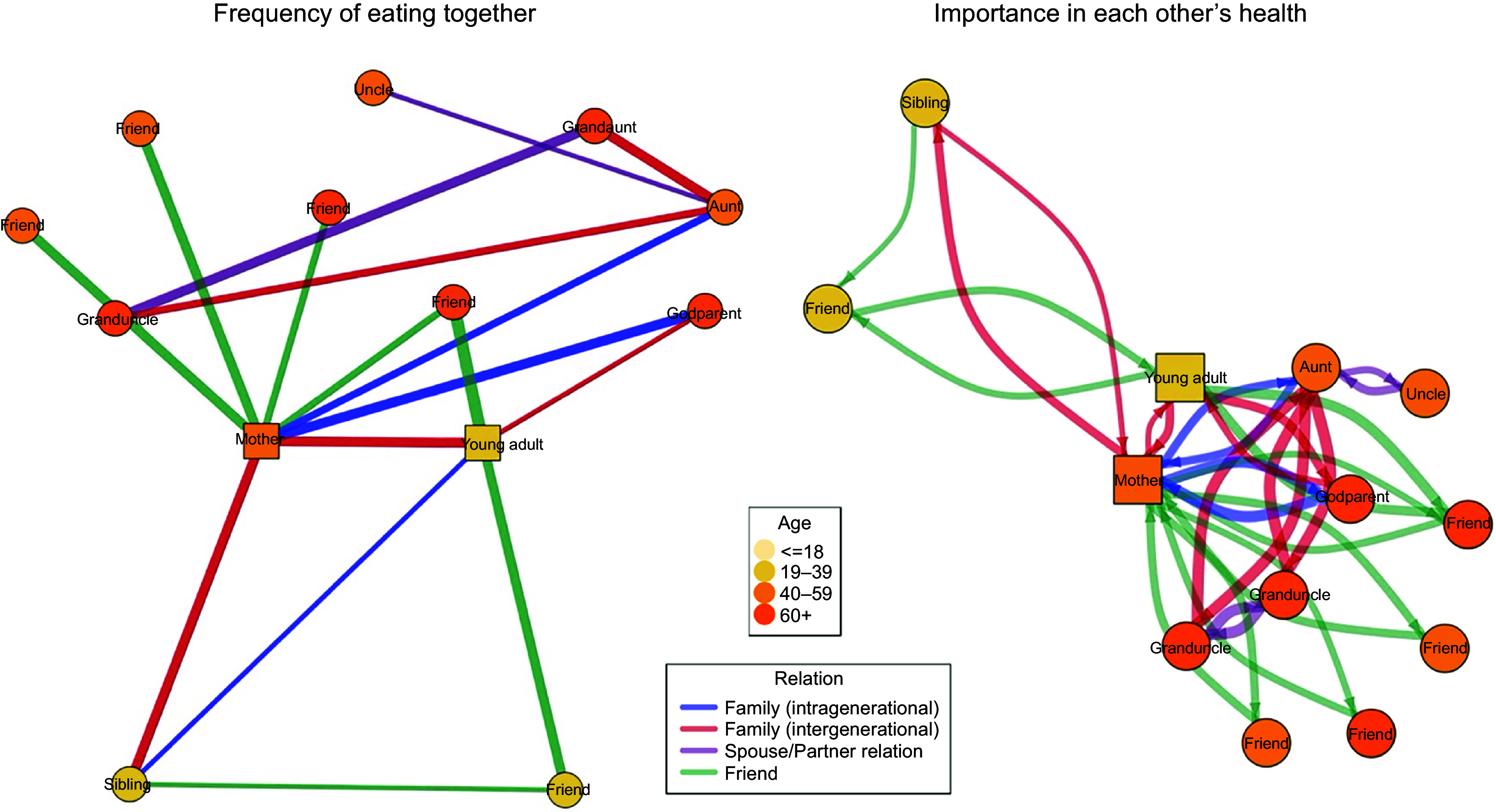
Example social network map of family unit analysed (13 nodes identified by 2 participants: young adult and mother). For ease in visual interpretability and exploring meaningful connections, only edges involving high (≥ 5 score) frequency of eating together or importance in each other’s health displayed. Edge thickness corresponds to frequency of eating together [left plot] and importance in each other’s health [right plot].

Network analyses of each household social network revealed a wide range of network sizes and structures (see online supplementary material, Supplemental File 1); an example of one analysed social network is visually displayed in Figure 2. Networks comprised an average of 9·7 nodes (sd: 4·7) and 33·2 edges (sd: 27·3). Assortativity analyses across all networks suggested that individuals of opposite sexes are more likely to eat with each other frequently (–0·19, sd: 0·17) and be important in each other’s health (–0·21, sd: 0·17). Moreover, individuals were also more likely to eat with and be important in the health of individuals who put a different level of effort (higher or lower) in their personal health (–0·20, sd: 0·21; –0·17, sd: 0·20).

**Table 1.** Characteristics of Singaporeans young adults and family members surveyed and others identified in networks (*n* 342)

Variable	Total (*n* 342)		Participants (*n* 116)		Others in Network (*n* 226)		*P* value
	*n*	%	*n*	%	*n*	%	
Age, *n* (%)							< 0·001
≤ 18	12	3·5	–		12	5·3	
19–39	173	50·6	71	61·2	102	45·1	
40–59	85	24·9	35	30·2	50	22·1	
≥ 60	72	21·1	10	8·6	62	27·4	
Sex, *n* (%)							0·556
Female	208	60·8	66	56·9	142	62·8	
Male	134	39·2	50	43·1	84	37·2	
	Mean	sd	Mean	sd	Mean	sd	
Health status, mean (sd)	6·4	1·7	7·1	1·5	6·1	1·7	0·764
Effort put in health, mean (sd)	5·9	2·0	6·6	1·8	5·6	2·0	0·958
	*n*	%	*n*	%	*n*	%	
Relation to young adult, *n* (%)							
Young adult	36	10·5	36	31·0	–		
Mother	35	10·2	27	23·3	8	3·5	
Father	26	7·6	17	14·7	9	4·0	
Sibling	46	13·5	26	22·4	20	8·8	
Aunt	24	7·0	–		24	10·6	
Uncle	22	6·4	1	0·9	21	9·3	
Cousin	17	5·0	5	4·3	12	5·3	
Grandparent	29	8·5	–		29	12·8	
Granduncle	1	0·3	–		1	0·4	
Grandaunt	2	0·6	–		2	0·9	
Spouse/Partner	4	1·2	3	2·6	1	0·4	
In-laws (Parent)	3	0·9	–		3	1·3	
In-laws (Sibling)	13	3·8	1	0·9	12	5·3	
Godparent	2	0·6	–		2	0·9	
Daughter	1	0·3	–		1	0·4	
Niece/Nephew	3	0·9	–		3	1·3	
Friend	78	22·8	–		78	34·5	
Race, *n* (%)							
Chinese	–		89	76·7	–		
Indian	–		17	14·7	–		
Malay/Other	–		10	8·6	–		
Household income, *n* (%)							
$2000–$5999	–		23	20·5	–		
$6000–$9999	–		46	41·1	–		
≥ $10 000	–		43	38·4	–		
Type of housing, *n* (%)							
1–3 room HDB	–		16	13·8	–		
4-room HDB	–		26	22·4	–		
5-room/Exec HDB	–		52	44·8	–		
Condominium/Landed	–		22	19·0	–		
Relationship status, *n* (%)							
Not Partnered or Married	–		56	49·6	–		
Partnered or Married	–		57	50·4	–		
	Mean	sd	Mean	sd	Mean	sd	
Individualism (4–36), mean (sd)	–		27·49	4·99	–		
Collectivism (4–36), mean (sd)	–		27·21	6·28	–		
BMI, mean (sd)	–		22·67	3·70	–		
	*n*	%	*n*	%	*n*	%	
Vegetable consumption							
4 or more times each day	–		2	1·7	–		
3 times each day	–		5	4·3	–		
2 times each day	–		25	21·6	–		
1 time each day	–		31	26·7	–		
4–6 times in the past week	–		28	24·1	–		
1–3 times in the past week	–		25	21·6	–		
None	–		–				
Fruit consumption							
4 or more times each day	–		2	1·7	–		
3 times each day	–		1	0·9	–		
2 times each day	–		5	4·3	–		
1 time each day	–		28	24·1	–		
4–6 times in the past week	–		28	24·1	–		
1–3 times in the past week	–		44	37·9	–		
None	–		8	6·9	–		
Fast food consumption							
4 or more times each day	–		–		–		
3 times each day	–		–		–		
2 times each day	–		–		–		
1 time each day	–		–		–		
4–6 times in the past week	–		7	6·0	–		
1–3 times in the past week	–		71	61·2	–		
None	–		38	32·8	–		
Snack consumption							
4 or more times each day	–				–		
3 times each day	–		2	1·7	–		
2 times each day	–		3	2·6	–		
1 time each day	–		13	11·2	–		
4–6 times in the past week	–		16	13·8	–		
1–3 times in the past week	–		74	63·8	–		
None	–		8	6·9	–		

HDB, Housing Development Board.

Adjusted analyses of personal and interaction characteristics associated with increased importance in a target node’s health are displayed in Table 2. In Model 1, both greater interactional frequency (0·21, 95 % CI: 0·16, 0·26) and emotional closeness (0·45, 95 % CI: 0·40, 0·49) were associated with an increased importance in a target node’s health. However, interaction frequency was observed to be no longer statistically significant when accounting for frequency of eating (0·39, 95 % CI: 0·35, 0·43) (Model 2). In both models, the amount of effort that the source node puts into their own health contributed to the degree of importance in the target node’s health. Spouse/partner and intergenerational family relations involved a significantly higher degree of importance in a target node’s health compared with friend relations. Sensitivity analyses were also conducted for both Models 1 and 2 among only network members who were directly surveyed in the study, and consistent findings were observed. When examining the role of individualism and collectivism in importance in a target node’s health (only assessed among surveyed participants), no significant association between either variable was observed (Model 3).

**Table 2.** Personal and interaction characteristics associated with increased importance in a target node’s health

	Model 1 (*n* 2242)*		Model 2 (*n* 2242)*		Model 3 (*n* 836)^†^	
	Interaction Freq + Emotional Closeness		Model 1 + Frequency eating		Model 2 + Individualism/Collectivism	
	*β*	95 % CI	*β*	95 % CI	*β*	95 % CI
Variable						
Interaction frequency	**0·21**	**0·16, 0·26**	0·03	−0·02, 0·08	−0·01	−0·09, 0·08
Emotional closeness	**0·45**	**0·40, 0·49**	**0·33**	**0·29, 0·38**	**0·38**	**0·30, 0·46**
Frequency of eating together	–	–	**0·39**	**0·35, 0·43**	**0·43**	**0·36, 0·49**
Individualism (source node)	–	–	–	–	0·04	−0·21, 0·30
Collectivism (source node)	–	–	–	–	0·22	−0·07, 0·52
Type of relation						
Friend	Ref	Ref	Ref	Ref	Ref	Ref
Family (intergenerational)	**0·86**	**0·66, 1·06**	**0·64**	**0·46, 0·83**	**0·62**	**0·29, 0·94**
Family (intragenerational)	**0·51**	**0·30, 0·71**	**0·44**	**0·25, 0·63**	**0·36**	**0·03, 0·68**
Family (spouse/partner)	**1·51**	**1·21, 1·81**	**0·90**	**0·62, 1·19**	**0·89**	**0·42, 1·35**
Age (source node)						
≤ 18	Ref	Ref	Ref	Ref		
19–39	0·31	−0·09, 0·7	0·32	−0·03, 0·68	Ref	Ref
40–59	0·31	−0·1, 0·72	0·33	−0·04, 0·70	0·12	−0·18, 0·43
60+	0·28	−0·16, 0·72	**0·44**	**0·05, 0·84**	0·07	−0·40, 0·53
Sex (source node)						
Female	Ref	Ref	Ref	Ref	Ref	Ref
Male	−0·08	−0·23, 0·07	−0·10	−0·23, 0·03	0·04	−0·19, 0·27
Health status (source node)	−0·06	−0·12, 0·00	−0·04	−0·10, 0·01	−0·09	−0·19, 0·01
Effort put into health (source node)	**0·12**	**0·07, 0·17**	**0·09**	**0·04, 0·13**	**0·10**	**0·02, 0·19**
Age (target node)						
≤ 18	Ref	Ref	Ref	Ref	Ref	Ref
19–39	**0·45**	**0·05, 0·85**	**0·46**	**0·10, 0·81**	**0·73**	**0·19, 1·28**
40–59	**0·49**	**0·08, 0·91**	**0·51**	**0·14, 0·89**	**0·70**	**0·12, 1·27**
60+	**0·46**	**0·02, 0·91**	**0·62**	**0·22, 1·02**	**0·88**	**0·27, 1·49**
Sex (target node)						
Male	Ref	Ref	Ref	Ref	Ref	Ref
Female	−0·05	−0·19, 0·10	−0·06	−0·20, 0·07	−0·11	−0·32, 0·10
Health status (target node)	**0·12**	**0·07, 0·17**	**0·09**	**0·05, 0·14**	**0·08**	**0·01, 0·15**
Effort put into health (target node)	−0·06	−0·12, 0·01	−0·04	−0·09, 0·02	−0·01	−0·10, 0·08

*Each of the 1145 analysed ties included two directed data points (2290), although forty-eight datapoints had missing data on health status (24) and effort put into health (24). ^†^Only includes interactions in which the source node was direct survey participant (and subsequently completed the individualism/collectivism scale). Bolded values indicate statistically significant associations (p < 0.05).

**Table 3.** Adjusted* associations between network characteristics and consumption of vegetables, fruits, fast food and snacks

	Vegetables (+2 times/day)		Fruits (+1 times/day)		Fast food (any)		Snacks (+4–6 times/week)	
	AOR	95 % CI	AOR	95 % CI	AOR	95 % CI	AOR	95 % CI
Full network*								
Avg. health status	1·16	0·58, 2·31	1·23	0·43, 3·52	0·97	0·45, 2·11	**3·80**	**1·64, 8·81**
Avg. effort put in health	0·63	0·35, 1·14	1·49	0·78, 2·85	0·49	0·24, 1·00	**0·51**	**0·28, 0·95**
Avg. frequency of eating together	0·94	0·70, 1·26	0·79	0·58, 1·07	1·07	0·77, 1·48	0·87	0·62, 1·23
Family^†^								
Avg. health status	0·99	0·55, 1·8	1·52	0·70, 3·30	1·03	0·53, 2·00	**3·21**	**1·58, 6·52**
Avg. effort put in health	0·81	0·53, 1·25	0·90	0·54, 1·51	**0·41**	**0·22, 0·77**	**0·50**	**0·29, 0·85**
Avg. frequency of eating together	0·86	0·66, 1·13	0·92	0·66, 1·30	1·39	0·97, 1·98	0·95	0·65, 1·40
Friends^‡^								
Avg. health status	1·91	0·57, 6·33	3·29	0·93, 11·72	2·43	0·84, 6·99	1·85	0·54, 6·37
Avg. effort put in health	0·57	0·24, 1·34	1·62	0·57, 4·59	1·23	0·52, 2·94	1·72	0·59, 5·05
Avg. frequency of eating together	1·27	0·81, 2·00	1·20	0·73, 1·97	**0·50**	**0·30, 0·84**	1·16	0·90, 1·50

*Adjusted for participant age, sex, race, income, BMI, self-rated health and effort put into one’s own health. ^†^For full network, 109 participants with complete, excluding those missing income (4), self-rated health and/or effort put into health (2) and BMI (1). For family network, 108 participants with family members in network (also excluding one participant without a non-spousal family member in their network). ^‡^For friend network, sixty-three participants (also excluding forty-six participants without friends identified in network). Bolded values indicate statistically significant associations (p < 0.05).

Adjusted analyses of the role of full, family and friend network characteristics and dietary behaviours revealed significant associations for health status, effort in health and frequency of eating together (Table 3). Higher average health status among the full network and family members in the network specifically were linked to increased snack consumption (AOR: 3·80, 95 % CI: 1·64, 8·81; AOR: 3·21, 95 % CI: 1·58, 6·52), but not to vegetable, fruit or fast-food intake. Greater average effort in health among network members was associated with lower snack consumption (AOR: 0·51, 95 % CI: 0·28, 0·95) and, among only family network members, both lower snack (AOR: 0·50, 95 % CI: 0·29, 0·85) and fast-food consumption (AOR: 0·41, 95 % CI: 0·22, 0·77). Additionally, eating more frequently together with friends was linked to reduced fast-food intake (AOR: 0·50, 95 % CI: 0·30, 0·84), though no significant associations were observed for vegetable, fruit or snack consumption.

## Discussion

This study provides valuable insights into the role of social networks in personal health and shared health behaviours (notably eating behaviours), highlighting the significant roles of emotional closeness, interaction frequency and shared meals in fostering reciprocal health importance, while suggesting a limited independent effect of greater adherence to cultural values like individualism and collectivism. By examining these dynamics within the culturally diverse context of Singapore, the findings underscore the importance of relational and interactional factors in health promotion. These results offer critical implications for designing interventions that leverage the strengths of both family and friend networks, particularly as social dynamics shift with urbanisation and changing socio-economic conditions.

The lack of significant associations between individualism or collectivism and mutual health importance or frequency of eating together is a notable finding, particularly given the theoretical relevance of these cultural constructs in shaping social behaviours^(19,21,41)^. One possible explanation is that while individualism and collectivism provide broad frameworks for understanding societal values, their influence may be less pronounced in specific, day-to-day family dynamics. Instead, more immediate relational factors, such as emotional closeness or practical caregiving expectations or responsibilities, may hold greater sway over behaviours like eating together or influencing each other’s health^(42)^. This finding suggests that the quality of individual relationships, rather than overarching value systems, plays a much more powerful role in shaping mutual health importance. For research and practice, this underscores the need to evaluate individual dyadic relationships more carefully rather than relying on assumptions based on prevalent values and beliefs when designing family-based or peer-led interventions. Additionally, updated qualitative research is needed to disentangle the contemporary role that individualistic and collectivist value systems play in shaping the health behaviours of Asian populations. This is especially relevant in the context of greater urbanisation and a changing socio-economic landscape, which are likely to reshape traditional values and social norms in ways that influence health behaviours and relationships^(19,21)^.

Family relationships demonstrated significantly greater reciprocal health importance than friend relationships, even after accounting for interaction frequency and emotional closeness. This distinction likely reflects the enduring nature of familial bonds, which are often strengthened by a sense of obligation, shared history and long-term commitment, in contrast to the voluntary and potentially less consistent nature of friendships^(43)^. Spousal/partner ties exhibited the highest frequency of eating together and reciprocal health influence, reflecting their unique intensity likely driven by cohabitation, shared routines and mutual caregiving^(44)^. Intergenerational relationships followed in importance, with their significance remaining robust even after adjustments, highlighting their complementary dynamics: older generations may provide guidance and mentorship, while younger generations may offer proactive support and access to resources^(43,45)^. These findings, consistent with prior research that documents frequent interaction and strong emotional bonds in intergenerational ties^(43,45)^, align with the rationale for involving adult children in chronic disease prevention and management interventions for older adults^(46)^. Our results, grounded in Family Systems Theory, underscore the interdependence and ripple effects of behaviours within family units, illustrating how individuals’ roles and actions influence the health and well-being of the entire family system. While friend-based interventions remain relevant^(47)^, family-based approaches (particularly those targeting spousal and intergenerational ties) may better leverage these dynamics. However, the ability to leverage these relationships may also depend on which health outcomes or behaviours are being targeted; further research is needed to better disentangle the specific health domains in which both family members and friends can be influential (especially in Singapore and other Asian settings).

A striking finding from our study was that individuals who perceived their social circles as being in better overall health were actually more likely to consume unhealthy snack foods. Understanding this pattern requires distinguishing between health status and health-promoting behaviours – two related but distinct concepts. Health status refers to the actual state of an individual’s well-being, whereas health-promoting behaviours reflect the willingness and ability to engage in actions that support better health. While having friends and family who do not engage in health-promoting behaviours has been linked to poorer health outcomes^(48)^, research also suggests that witnessing health deterioration within one’s social network can serve as a wake-up call, prompting individuals to adopt healthier behaviours^(48,49)^. In contrast, when those around us appear healthy, there may be less perceived urgency to make proactive health choices, leading to a false sense of security and, consequently, greater indulgence in unhealthy foods. Notably, we found no association between perceived social health and the consumption of fruits and vegetables. This suggests that the influence of social health may operate primarily as a warning mechanism to discourage unhealthy eating, rather than as a source of motivation to adopt healthier dietary habits.

Complementing the aforementioned findings related to health status, our results also highlight the role of effort in maintaining health. Specifically, individuals consumed fewer unhealthy snacks when their social networks demonstrated greater efforts towards health. This aligns with research showing that individuals surrounded by health-conscious relations are more likely to engage in healthy practices themselves, as increased opportunities for bidirectional influence through various verbal and non-verbal cues are more pertinent, likely taken advantage of, and easier to implement or sustain^(45)^. Snacking, in particular, is often a communal activity, with Singaporean young adults frequently sharing unhealthy snacks in social settings^(14)^. However, when health is prioritised within a social circle, the availability and accessibility of such foods likely decrease, leading to lower consumption. Notably, as with health status, these associations were observed only in relation to unhealthy dietary behaviours such as fast food and snacking, with no corresponding increase in healthy behaviours like fruit and vegetable consumption. This reinforces the idea that the protective effects of social networks on diet function primarily by discouraging unhealthy eating rather than actively promoting healthier dietary habits.

Lastly, the reduced fast-food consumption associated with eating out with friends can be partly explained by Singapore’s unique food culture. The widespread availability of hawker centres and food courts that offer a diverse range of cuisines at prices comparable to or even lower than some traditional fast-food outlets makes these options more appealing, particularly when convenience and affordability are prioritised^(50)^. Additionally, such venues better accommodate diverse palates and dietary restrictions, key factors in group decision-making when selecting a place to eat. Qualitative interviews conducted with a subset of surveyed participants, described in a separate study (under review), further suggest that lunch breaks with coworkers constitute a major context in which these ‘meals with friends’ occur. In Singapore, hawker centrers and workplace canteens have been strategically developed near office hubs to cater to this type of mealtime setting, reinforcing their role as preferred dining locations^(50)^. These findings underscore the need for policy efforts aimed at leveraging social influences on dietary behaviours to account for local cultural and environmental contexts and how different communities may define ‘fast-food’. Assumptions about how social influences shape eating habits may not hold across different settings, and interventions must be tailored to the specific dynamics of where and how meals with friends and family are consumed.

The study’s strengths include its innovative use of SNA and multilevel modelling to examine family and peer dynamics, its focus on the understudied cultural context of Asia and its comprehensive mapping of relational characteristics such as emotional closeness, interaction frequency and shared meals. However, several limitations should be acknowledged. First, the recruitment strategy, which relied on online outreach, may have introduced selection bias, potentially limiting generalisability to less digitally connected populations. Additionally, all analysed networks included a young adult (as the study was nested within a larger young adult intervention), and network size was constrained by the number of individuals participants were able and willing to identify. While most participants provided clarification on ambiguous nodes, in cases where they did not, estimations had to be made through triangulation, which carries a risk of error. Nonetheless, the study’s ability to analyse diverse family structures and triangulate node and relational data from multiple family members was a strength. Future research should further expand network diversity, conduct more exhaustive assessments of family and friend networks and enhance methods for confirming node identities and relationships.

Moreover, the study’s reliance on self-reported measures, such as emotional closeness and frequency of eating together, may have been subject to social desirability and recall bias. The brief measures of dietary behaviour and self-rated effort may also be prone to potential measurement error and do not capture the full complexity of dietary or health behaviours. Additionally, participants’ reports on the dynamics between identified important persons who were not participants were based on their own perceptions and may be difficult to verify, though the study’s ability to triangulate and average data on some of these individuals from multiple participants helped mitigate this limitation. Likewise, using relative rather than specific or objective measures (e.g. visit frequency or specific health behaviours) facilitated reflection on network dynamics via survey but may limit precision; while the individualism/collectivism items showed acceptable internal consistency in this sample and have been used in other Asian contexts^(31,32)^, they too may carry potential measurement error and have not been specifically validated for Singapore. Scaled-up research could benefit from more intensive network assessments incorporating additional data sources or direct consultation with network members. Third, the cross-sectional design prevents causal inferences. Finally, the limited sample size, particularly among older generations and non-family peers (as analyses were nested within a larger study centered on family relationships), may have constrained the detection of subtle interaction patterns or demographic differences. Future research should employ longitudinal designs to establish causality, integrate more diverse or objective measures of health behaviours and family dynamics and recruit larger, more diverse samples to enhance generalisability and deepen insights into social networks across different demographic and cultural contexts.

Overall, this study underscores the critical influence of social networks, particularly family relationships, in personal health and shared eating behaviours. The findings emphasise the unique role of shared meals and emotional closeness as key mechanisms driving mutual health influence, offering actionable insights for designing culturally resonant and family-involved interventions. As urbanisation and socio-economic changes continue to reshape traditional social dynamics, future efforts must focus on leveraging these relational strengths to promote sustainable health behaviour change. Building on this foundation, future observational research should use larger, diverse samples and longitudinal study designs to map how specific network structures (such as relational strength and network density) interact with urban living patterns, work demands and family obligations to influence dietary and lifestyle choices over time. In-depth qualitative studies could further unpack how cultural norms, household roles and changing economic pressures shape the specific mechanisms by which family and peer ties affect daily health practices. In parallel, intervention research can test practical ways to mobilise family and peer networks, such as involving household members in shared goal setting and co-developing supportive routines that fit contemporary urban contexts. Policy and practice should also consider embedding family- and network-based strategies into workplace wellness, community nutrition programs and primary care, creating environments that strengthen positive social influence while addressing structural barriers to healthy living.

## Supporting information

Ali et al. supplementary material Ali et al. supplementary material

## Supplementary material

For supplementary material accompanying this paper visit https://doi.org/10.1017/S136898002510133X
