# Supplementary material for: Applying network analysis to explore family and peer influence on diet and health in households of Singaporean young adults: findings from an online network survey
Source: Public Health Nutr. 2025 Nov 14;28(1):e183. doi: 10.1017/S136898002510133X (PMC12722069; doi:10.1017/S136898002510133X)
Supplement: Ali et al. supplementary material [file S136898002510133Xsup001.docx]

| **Supplemental File 1: Characteristics of family networks of Singaporeans survey respondents (n=116)** | | | | | | | |  |  |  |
| --- | --- | --- | --- | --- | --- | --- | --- | --- | --- | --- |
|  |  |  | **Assortivity in frequency of eating together** | | | | **Assortivity in importance in each other’s health** | | | |
| **Family** | **Total nodes** | **Total edges** | Age | Sex | Health status | Health effort | Age | Sex | Health status | Health effort |
| Mean (SD) | 9.7 (4.7) | 33.2 (27.3) | -0.09 (0.17) | -0.19 (0.17) | -0.12 (0.24) | -0.2 (0.21) | -0.09 (0.17) | -0.21 (0.17) | -0.09 (0.23) | -0.17 (0.2) |
| Range | [4, 20] | [6, 101] | [-0.45, 0.38] | [-0.57, 0.2] | [-0.57, 0.42] | [-0.6, 0.24] | [-0.44, 0.28] | [-0.61, 0.11] | [-0.49, 0.4] | [-0.53, 0.28] |
| H01 | 13 | 100 | -0.01 | -0.14 | -0.12 | 0.11 | -0.03 | -0.09 | -0.18 | 0.09 |
| H02 | 13 | 120 | 0.03 | 0.05 | 0.41 | 0.03 | 0.02 | 0.03 | 0.37 | 0.04 |
| H03 | 6 | 24 | -0.27 | -0.32 | 0.10 | -0.01 | -0.30 | -0.39 | 0.22 | -0.08 |
| H04 | 16 | 160 | 0.41 | 0.01 | -0.07 | -0.29 | 0.22 | -0.18 | -0.07 | -0.24 |
| H05 | 6 | 30 | -0.29 | -0.15 | -0.34 | -0.02 | -0.37 | -0.08 | -0.18 | -0.13 |
| H06 | 8 | 44 | -0.08 | -0.17 | -0.15 | -0.18 | -0.13 | -0.15 | -0.22 | -0.17 |
| H07 | 6 | 30 | -0.16 | -0.26 | -0.20 | -0.24 | -0.18 | -0.16 | -0.19 | -0.38 |
| H08 | 14 | 126 | 0.08 | -0.18 | -0.21 | -0.17 | -0.02 | -0.13 | -0.22 | -0.32 |
| H09 | 9 | 72 | -0.05 | -0.23 | -0.18 | -0.13 | -0.18 | -0.16 | -0.19 | -0.20 |
| H10 | 7 | 34 | -0.08 | -0.20 | -0.25 | -0.40 | 0.07 | -0.40 | -0.44 | -0.32 |
| H11 | 17 | 80 | 0.06 | 0.14 | 0.44 | 0.10 | 0.08 | 0.12 | 0.42 | 0.07 |
| H12 | 9 | 26 | 0.45 | -0.20 | -0.04 | 0.10 | 0.48 | -0.25 | -0.17 | -0.10 |
| H13 | 10 | 52 | -0.22 | -0.24 | 0.30 | 0.22 | -0.13 | -0.22 | 0.30 | 0.28 |
| H14 | 13 | 108 | 0.01 | -0.10 | 0.10 | 0.06 | 0.06 | -0.12 | 0.34 | 0.16 |
| H15 | 17 | 164 | -0.03 | 0.00 | 0.01 | -0.17 | -0.04 | 0.03 | 0.06 | -0.17 |
| H16 | 8 | 50 | -0.16 | -0.15 | -0.16 | -0.15 | -0.17 | -0.15 | -0.17 | -0.16 |
| H17 | 5 | 14 | -0.28 | -0.39 | -0.49 | -0.51 | -0.32 | -0.54 | -0.55 | -0.45 |
| H18 | 6 | 28 | -0.10 | -0.37 | -0.23 | -0.40 | -0.11 | -0.41 | -0.14 | -0.42 |
| H19 | 7 | 36 | -0.01 | -0.24 | -0.23 | -0.35 | 0.01 | -0.32 | -0.14 | -0.33 |
| H20 | 17 | 202 | -0.08 | -0.15 | -0.06 | -0.19 | -0.11 | -0.18 | -0.05 | -0.19 |
| H21^1^ | 5 | 18 | - | -0.57 | -0.05 | 0.00 | - | -0.72 | 0.17 | 0.09 |
| H22 | 5 | 16 | 0.20 | -0.60 | -0.33 | -0.44 | -0.02 | -0.56 | -0.25 | -0.21 |
| H23 | 20 | 170 | 0.17 | -0.09 | 0.25 | 0.08 | 0.21 | -0.10 | 0.39 | 0.25 |
| H24 | 16 | 144 | 0.23 | 0.19 | 0.09 | 0.01 | 0.29 | -0.05 | -0.11 | 0.05 |
| H25 | 4 | 12 | -0.32 | -0.36 | -0.36 | -0.36 | -0.36 | -0.34 | -0.34 | -0.34 |
| H26 | 9 | 38 | -0.09 | -0.26 | -0.05 | -0.08 | -0.11 | -0.22 | 0.07 | -0.01 |
| H27 | 8 | 48 | -0.15 | -0.20 | -0.07 | -0.29 | -0.17 | -0.14 | -0.08 | -0.31 |
| H28 | 7 | 30 | -0.17 | 0.03 | 0.08 | 0.17 | -0.17 | -0.06 | 0.04 | 0.11 |
| H29 | 9 | 62 | -0.17 | -0.23 | -0.15 | -0.08 | -0.19 | -0.12 | -0.09 | -0.04 |
| H30 | 4 | 12 | -0.50 | -0.53 | -0.49 | -0.53 | -0.50 | -0.45 | -0.43 | -0.45 |
| H31^1^ | 5 | 16 | -0.19 | - | -0.13 | -0.13 | -0.25 | - | -0.02 | -0.02 |
| H32 | 8 | 50 | -0.15 | -0.31 | -0.14 | -0.06 | -0.15 | -0.29 | -0.23 | -0.15 |
| H33 | 7 | 38 | 0.03 | -0.42 | -0.16 | -0.26 | 0.05 | -0.37 | -0.15 | -0.22 |
| H34 | 10 | 32 | 0.23 | -0.29 | 0.01 | -0.39 | 0.26 | -0.32 | 0.06 | -0.38 |
| H35 | 9 | 48 | -0.30 | -0.13 | -0.28 | -0.19 | -0.21 | -0.11 | -0.26 | -0.17 |
| H36 | 9 | 56 | 0.15 | -0.19 | -0.09 | 0.29 | 0.06 | -0.46 | -0.13 | 0.14 |

^1^ No variability in either age or sex among the important people identified among the participants in this household
